# The *Noctambuli*: tales of sleepwalkers and secrets of the body in seventeenth-century England

**DOI:** 10.1080/0268117X.2020.1855235

**Published:** 2020-12-17

**Authors:** Elizabeth Hunter

**Affiliations:** Queen Mary, The University of London, London

**Keywords:** Sleepwalking, dreams, wonder, witchcraft, secrets

## Abstract

Seventeenth-century readers were fascinated by marvellous tales of people known as the *noctambuli* who rose up in their sleep, performed daily chores and attempted dangerous feats, such as clambering onto rooftops. On account of their uncanny nature, commentators noted that sleepers could be mistaken for spectres or those who had been bewitched. Depictions of the *noctamubli* were also influenced by the *Malleus Maleficarum*, which had argued that they were acting under the influence of demons and would fall if their Christian name was called. Medical texts explained this behaviour in terms of the escape of hot vapours within the body, the powers of the imaginative faculty, and the impairment of common sense during sleep. While this explanation was widely accepted, in the 1650s it was challenged by alternative views from esoteric writings, which conceptualised the movements of sleepers in terms of mystical powers within the body.

## Introduction

In 1699 John Dunton, bookseller and editor of the popular gentleman’s magazine *The Athenian*, recorded a conversation between himself and two friends that had taken place during a visit to a private house in Dublin the year before. By the warmth of a good fire one evening they “fell into a pleasant Chat” about “the *Antipathies in Nature*”, such as the author’s strong aversion to cheese, which had almost led to his death as a young child, and the case of a man who had fallen into a death-like swoon upon putting on a muff made of cat fur:

This led us to talk of *Sympathy*, and the Wonders thereof; and more particularly of Sir *Kenelm Dighy’s [sic] Sympathetical Powder*, and the great Cures wrought by it. From hence we fell to talk of a third Wonder of Nature, viz. *Mens walking in their Sleep*; of which Mr. *Larkin* gave a memorable Relation of a House supposed to be haunted; which was only occasioned by one of the Gentlemans Daughters, *who walked in her Sleep every Night*; which was at last discover’d by a Stranger’s having Courage enough to lie in the Room said to be haunted: This naturally led us in the fourth place, to talk of *Apparitions*; and here Mr. *Harman* ask’d me what I thought of a *Spectrum*’s assuming a *Humane Shape*? I assur’d him they might. [*The gentlemen then share a number of anecdotes of ghostly appearances, to which Mr. Larkin adds*] That he look’d upon the *denying of Spirits and their appearing to Persons after Death, to be the next degree to Atheism*.^1^

This casual conversion about strange diseases, marvellous cures and haunted houses provides an insight into the cultural history of sleepwalking in premodern England. In recent years research by William MacLehose and Sasha Handley has greatly advanced our understanding of this much understudied subject. MacLehose has shown that by the fourteenth century, physicians had developed a sophisticated explanation for sleepwalking in terms of how the brain behaved during sleep, when reason and external sensation were disabled. Unfettered by these regulatory functions the imagination presented images to the sleeper’s mind to which he often reacted violently, imagining he was hunting or fighting. This rendered the medieval sleepwalker a dangerous figure. Moving forward four centuries, Handley explains how Thomas Willis’s new theories of brain function led in the eighteenth century to the creation of a new category of nervous disorder called *somnambulism* – one of a number of fashionable ailments that became part of the cult of sensibility.^2^

These studies suggest that perceptions of sleepwalking tend to reflect the cultural preoccupations of the time. The underlying question remained the same: how could people who were asleep move around as though they were awake? The contexts in which this question was discussed were markedly different. In medieval medicine, the sleepwalker’s actions were regarded as an expression of the habits, desires and anxieties of the day surfacing during sleep.^3^ In the eighteenth century, the neurological view of sleepwalking as a symptom of nervous activity in the brain gave rise to the notion that sleepwalkers possessed particularly penetrating powers of perception.^4^ The issues that the gentlemen raise in their discussion in the late seventeenth century position sleepwalking in another context. Here the behaviour of sleepers is significant in terms of what it could suggest about cosmological forces of sympathy and antipathy or the existence of personal spirits. This places it within the history of magic.

The historiography on the history of magic and science has shown that during the sixteenth and seventeenth centuries there was heightened interest in accounts of unusual and strange phenomena that existed on the contested boundary between the natural and the supernatural realm. Michael Hunter argues in his recent monograph *The Decline of Magic: Britain in the Enlightenment* (2020) that this interest became particularly intense in intellectual circles in England in the late seventeenth century as prominent figures connected to the Royal Society searched for evidence to counter the perceived threat from materialist atheism.^5^ Sleepwalking belonged in this category of strange and wonderful events, sometimes referred to as the preternatural. This article examines sleepwalking in this context, showing how debates over the existence of the supernatural provided a framework for interpreting the amazing tales of sleepwalkers found in wonder books from this period.

That sleepwalking should have been thought of as a liminal state on the edge of wonder is unsurprising considering the number of studies from this period suggesting a close connection between sleep and the supernatural realm. Keith Thomas included a short section on prophetic dreams in *Religion and the Decline of Magic*.^6^ Since then, studies of dreams have shown a strong connection between the experiences of sleepers and both belief in the supernatural realm and sceptical arguments against it. It was in dreams that people received visits from dead relatives, met with angels or the devil, were transported to Heaven or Hell, and had premonitions of their own death or the death of family members. Many of these dreams were politically significant, and this is one reason that debates over the meaning of dreams – or whether they were meaningful at all – became fraught, particularly in the post-Civil War period. Humoral explanations that saw dreams as “nothing els but a bubling scum or froath of the fancie, which the day hath left undigested” (in the memorable words of Thomas Nashe) were important in undermining the authority of prophetic dreaming as well as belief in the interference of dark spirits.^7^ Sleep continues to be an important element in sceptical approaches today as historians study modern dream theory to discover explanations for the vibrant faith in the supernatural world evident in early modern culture. Over the past two decades Emma Wilby and Edward Bever have used insights from cross-cultural or scientific investigations of dreams to account for fairy beliefs and testimonies of attendance at black sabbath rituals, both in Britain and Europe.^8^

Sleep studies therefore have much to offer in terms of our understanding of the rise and decline of supernatural systems of thought. However, what would in modern terms be called sleep disorders have received much less attention than dreaming. The few studies that there are focus mainly on the incubus disease, which has parallels with the modern diagnostic category of sleep paralysis. Owen Davies first drew attention to the relationship between the symptoms of this disorder (known as the “mare” in English) and popular beliefs about the nightmare spirit in an article published in *Folklore* in 2003. He found substantial evidence linking witchcraft accusations in English trial records to experiences of sleep paralysis/nightmare.^9^ Janine Rivière includes a chapter on sleep disorders in her recent monograph *Dreams in Early Modern England* (2017). Here she argues that supernatural and natural explanations for sleep disorders co-existed well into the nineteenth century. While physicians attributed insomnia, oversleeping, bad dreams, starting out of sleep and the nightmare to physiological causes, such as a dry brain or an upset stomach, the belief that sleep problems were caused by witchcraft or demonic attack persisted amongst the general population.^10^

In this article, I will demonstrate that sleepwalking also featured in debates over the powers of demons, the distinction between bewitchment and disease, and the significance of dreams, although it has so far not been mentioned in historical studies of these issues. There was a difference between accounts of sleepwalking and accounts of dreams or the nightmare, which made the subject of particular interest to natural philosophers. Whereas evidence for dreams or attacks by night spirits was based on the recollections of sleepers, sleepwalkers did not remember how they had behaved during the night. Accounts of sleepwalking were based upon the observations of those awake and provided (by early modern standards) more objective evidence of what happened to the body and mind during periods of unconsciousness. Whether or not ghosts troubled the living, people received revelations in dreams, or witches travelled at night was a matter of debate; but that people got out of bed in their sleep, unlocked doors and climbed onto rooftops where they walked about was not. “The matter of Fact”, wrote Thomas Tryon in his *Treatise of Dreams and Visions*, “ … is so notorious, that I need not spend time to prove it by instances”. The question was, what was this evidence of? – “But the Cause or Reason of all this”, he added, “is more occult”.^11^ In this article I look at three possible interpretations of sleepwalking available in seventeenth-century England: the operation of demons, the effects of animal spirits in combination with the powers of the imaginative faculty, and the existence of an astral body or “intelligence” that became active during periods of unconsciousness. In the first half of the century, when sleepwalking was embroiled in debates over bewitchment and the powers of the imagination, the sleepwalker was conceptualised as a demoniacal-like figure that would suddenly fall down if their name was called, as though dispossessed. From the middle of the century a new visualisation of the sleepwalker emerged as an ecstatic figure, whose motions were evidence of a vital energy active within the sleeping body in the form of an astral body or bodily spirits receptive to premonitory dreaming.

## Transgressions of the night

Sleepwalking was not a term generally used in seventeenth-century England.^12^ English writers more commonly employed the Latin term *noctambulo* (combining *nox* [night] and *ambulate* [to walk, move about]), or its anglicised form “nightwalker”. In the eighteenth century, the preferred term became the Latin form of “sleepwalking” *somnambulism*. Handley sees this linguistic change as indicative of a shift in cultural perceptions of sleep. Whereas the approach to slumber in the previous century had been holistic, seeking the regulation of both body and soul, sleep problems were now regarded in primarily somatic terms.^13^ I would argue that it can, in addition, be explained in terms of the relative decline in respect for the sacred rhythms of day and night between the two centuries that meant that more people were socialising during night hours. For much of the seventeenth century society still retained the medieval view that regarded nightly movements with suspicion. The term “nightwalker” had a number of meanings that all referred to irregular activity. In legal terms, it was the appellation given to people found out on the streets after dark with no legitimate purpose.^14^ It had a third meaning in the context of witchcraft treatises, where the flight of witches was occasionally referred to as nightwalking; as, for instance, when Reginald Scot wrote that “Witches nightwalkings are but Phantasies and Dreams”.^15^

There was therefore a semantic connection between sleepwalkers and the nocturnal travels of witches. Further links between these two concepts will become apparent later in this article. Nightwalking in all three instances had connotations of transgression. To move about at night, whether asleep or awake, with or without supernatural aid, was considered a breach of the laws of nature and civil society. Sleep belonged to the night, movement to the day.^16^

It is useful to consider here that the early modern concept of the *noctambulo* and modern understandings of sleepwalking are not entirely synonymous. There is significant overlap between early modern descriptions of people moving around while asleep and the sleep arousal disorders sleepwalking and night terrors. The main feature of sleepwalking is complex motor behaviour initiated during sleep. After getting up out of bed and walking about, sleepwalkers may perform actions that are strange or out of place, such as urinating in a bin. During the episode their eyes are open with a blank stare and they are difficult to awaken. When they do wake up they typically remember nothing of the episode. An individual suffering from night terrors will sit up suddenly in bed and start screaming or crying. They display similar amnesia upon awakening.^17^ In unusual circumstances sleepwalkers have been known to perform surprisingly complex tasks, such as getting into a car and driving away. However, a main feature of early modern sleepwalkers was that their abilities went beyond what people would be able to accomplish when awake. Their agility was superhuman, enabling them to travel through normally impassable places, such as walking on rooftops or over narrow bridges.

In this article, I have avoided the term “nightwalker” because it could be confused with the legal meaning, but I use the term *noctambulo* to distinguish this more fantastical conception of sleepwalking from modern ideas about sleepwalking. Sleepwalkers were often referred to collectively, with the plural being formed in various ways – *noctambuli, noctambulones,* and *noctambulos*. I have adopted *noctambuli* for consistency.

## Wonderful histories

Tales of the *noctambuli* were part of the literature of wonders and prodigies that became popular in England over the course of the seventeenth century. These served a variety of purposes, from providing the reader with informative and entertaining conversation to supplying evidence of the providential hand behind natural, but awe-inspiring *miranda*.^18^ An example is Nathaniel Wanley’s *The Wonders of the Little World* (1673), a collection of thousands of examples from history, philosophy and medicine of marvellous and strange occurrences. The influence of this work can be seen in the conversation recorded by Dunton fifteen years later. It included a chapter on the “natural antipathies in some men, to flowers, fruits, flesh, physick, and divers other things”, with an anecdote attributed to Sir Kenelm Digby of a woman whose cheeks were blistered by a rose, and various cases of severe food allergies. Another chapter addressed the topic of apparitions, in which he wrote that those who disbelieved in demons and spectres were “men possessed with … uncurable madness”, and there were two chapters on wonderful sleepers, one recounting legends of people who had slept for marvellously long periods of time, and another “Of such men as have used to walk and perform other strange things in their Sleep”.^19^

By the time Wanley was writing, the canon of tales of the *noctambuli* had been greatly expanded by the publication of a number of continental collections of wonders. Most notably, Edward Grimestone’s influential translation of a compilation of marvels and providences by the French Calvinist Simon Goulart, published as *Admirable and Memorable Histories Containing the Wonders of our Time* (1607), contained a section on “Marveylous Sleepers”.^20^ Therefore, in addition to the medieval tales of aristocrats, lawyers, and poets who fought, debated and wrote in their sleep, Wanley also included tales of people who performed other complex daily tasks while asleep, such as washing or dressing, and who also attempted dangerous feats, such as clambering onto rooftops. There was also a new tale of a local man, John Poultney of little Sheepy in Leicestershire, who was reputed to rise out of bed in his sleep, dress himself and walk around the fields, which also appeared in George Sandy’s history of England in a section on “memorable persons”.^21^

Wonder books emphasised the marvellous nature of the *noctambuli*. Thomas Lupton in *A Thousand Notable Things* introduced his account of a person rising in their sleep as “marvellous straunge, and almost incredible”.^22^ Sleepers were able to accomplish the same tasks that they had completed during the day, such as dressing themselves, unlocking doors or navigating streets while still asleep. Women baked bread and arranged hair. A taverner ran up and down the streets at night as though mad, but was able to thwart thieves who tried to steal the keys to his wine cellar from his side. A man went up and down the stairs locking and unlocking his chests at night.^23^ A poet composed verses in his sleep, sometimes writing for up to four hours. Another played the lute.^24^

The *noctambuli* also displayed a recklessness that endangered themselves and others. Thomas Lupton’s *noctambulo* rose in his sleep and went into the woods where he killed a buck. He then returned to his chamber and thrust about the mattress with his sword, almost killing his bedfellow.^25^ Tales from other collections included one of a man who put on his boots and spurs, climbed out of a garret window and rode upon a washing pole hanging over the street, a student living in Paris who went out into the streets one night and killed a young child in his sleep, and a man who gave himself a near-mortal wound fighting an imaginary enemy. A popular tale, repeated in a number of collections, was of a student who fearlessly climbed onto a high roof in order to steal eggs from a magpie’s nest. An unfortunate accident befell two young men from Meissen, who would walk about on rooftops while asleep, when one fell off and broke his thigh. In Neapolis, a man fell out of a window and almost died of his injuries.^26^ This recklessness could also manifest itself in a disregard for the normal rules of decorum. A shopkeeper went through the streets stark naked to unlock the door of his shop. A young student of physic walked around his chamber undressed before climbing onto a high window sill. A young woman in Paris went every night unclothed through the streets to bathe in the river until her father found her and beat her.^27^ All this was done with apparently no sense of peril or embarrassment. Unless, like the young man who climbed onto the washing pole, and a gentleman who climbed down a well, the sleeper woke up in the middle of the episode, in which case they became ill with fright.^28^

It was not always clear whether the exploits of the *noctambuli* should be taken as fact or fiction. In a note to the reader of the English version of Goulart’s *Histories*, the printer advised “If any thinke that by the name of Histories, all should be true, he may knowe Historiographers confesse they may write as they list”. Thomas Lupton commented that the story of the sleepfighter was “a marvellous matter if it were true”.^29^ There is also the possibility that these incredible stories were not taken entirely seriously by some of their readers. Ben Jonson seemed to mock the genre in *The Magnetic Lady* (first performed in 1632). The play included a sleepwalker

That talked in’s sleep; would walk to Saint John’s Wood

And Waltham Forrest, scape by all the ponds

And pits i’the way; run over two-inch bridges;

With his eyes fast, and i’the dead of night!^30^

Most seventeenth-century commentators, however, accepted as fact that people in their sleep could perform amazing feats. Their concern was not with the truth of these histories, but with causation.

## The sleeping body in early modern medical theory

There is evidence of a close connection between the genre of wonder and medical discourse on sleep and sleep disturbances. As some stories of the *noctambuli* were taken from physicians’ writings, they retain traces of medical details on the case. A student in Goulart’s collection who climbed onto a high windowsill in his sleep was described as a “young man of lively spirit, small stature, and slender body, but of a moyst braine”.^31^ Similarly, medical texts from this time suggest the influence of wonder books, describing the behaviour of people who rose in their sleep in terms of marvel and curiosity. For instance, in *Klinike, or the Diet of the Diseased* (1633), a popular work promoting healthy living, the Northampton physician James Hart included a passage on sleepwalking, which began:

Now to give some satisfaction to the curious Reader, I will say something concerning a point depending upon the former: and that is concerning such as during their naturall sleepe, yet performe such actions as are commonly performed by such as are awake, to the no small astonishment and amazement of the beholders, and are called therefore *Noctambuli*, or night-walkers.

Hart gave a physiological explanation for this phenomenon. It occurs, he wrote, because during sleep the discerning faculty is overcome by the thick, misty vapours that rise up into the brain, causing the sleeper to take what he sees in dreams for reality and act upon these visions.^32^

According to early modern theories of the body, sleep occurred when vapours created in the stomach by the processes of digestion rose up into the head. The hot vapours upon reaching the cold moisture in the brain caused a kind of seizure in the body, in which the heat from the outer parts rushed towards the heart resulting in a cessation of the functioning of the outward senses.^33^ Occasionally some residual spirits escaped during this process into the outward parts of the body, and this was what caused sleepwalking. The key to explaining the actions of those who rose in their sleep was that, while the faculty of common sense was rendered inoperative by the vapours of sleep, the fantasy remained active, able to produce images which appeared to the sleeper in the form of dreams. It was by this guide that the sleepwalker moved about and executed tasks.

Hart therefore included his passage on the *noctambuli* in a chapter on dreaming, which he explained as a product of *species* left over from the day from which the “fancy” produced visions presented to the imagination.^34^ His was the classic Galenic explanation for the movements of sleepers, based in a materialist conception of dreaming found in philosophical and medical works across Europe at this time. The common sense (*sensus communis*), and fantasy (*phantasia*) were functions of the imagination, which was part of the sensitive soul, and therefore subject to the effects of bodily changes. The faculty of common sense was responsible for uniting sensations received from the five senses. It thus imposed order upon the images produced by the fantasy – or “fancy”, as it was sometimes known in English. When the vapours of sleep caused both the faculty of common sense and the outward senses to cease functioning, the fantasy continued to function, unrestricted by the operations of the common sense, and sealed off from information received from the external world during waking hours via the senses. It continued to present residual sense impressions (stored in the memory) to the imagination, which appeared in the form of dreams.^35^

### Led by the fiend

The view that the actions of sleepers were made possible by the escape of animal spirits and were guided by dreams was well established in Europe by this time, and can be traced back to Aristotle’s essay “On Sleep”.^36^ However, this somatic explanation took on a new significance in the late medieval and early modern period when the behaviour of the *noctambuli* became associated with demonical activity.

Prior to the seventeenth century, a number of prominent writers across Europe attributed the fearless or self-destructive behaviour of *noctambuli* to diabolical interference. This was imagined as a form of obsession, rather than possession: the sleeper’s actions were instigated by the suggestions of the devil, or their bodies were prevented from falling from great heights by the power of demons. Thomas More, for instance, suggested that the suicidal should be tied to the bed in case they were prompted by the devil to rise and hang themselves in their sleep.^37^ The Spanish Renaissance poet Antonio de Torquemada told the story of a man named Tapia who was led by a demon in his sleep to a fast-flowing river where he almost drowned. This tale appeared in both a 1600 English translation of Torquemada’s *Jardín de flores curiosas* and in Goulart’s *Memorable Histories*.^38^

The most significant writer to shape demoniacal depictions of the *noctambuli* across Europe at this time was the fifteenth-century Dominican inquisitor Henreich Kramer, author of the *Malleus Maleficarum* (“The Hammer of Witches”). Kramer referred to the *noctambuli* at two points in the *Malleus*. One was in a chapter on lawful forms of exorcism in which he considered whether it was proper to rebaptise bewitched persons. The other was in a chapter demonstrating the metaphysical possibility of the transportation of witches. He claimed that those who walked across tall buildings in their sleep without being harmed were supernaturally supported by the power of demons in the same way that witches were transported through the air to attend sabbatical gatherings. “The amazing thing is”, he wrote “that when they are referred to by their own names, they are suddenly dashed to the ground as if the name may not have been bestowed on them in the appropriate manner at their baptism”.^39^

For Kramer, the *noctambuli*’s fearless feats and their dramatic fall provided evidence of powerful demonic forces at work at night that were responsible for the displacement both of those who walked in their sleep and witches who were transported to sabbatical meetings. There is evidence that the belief that demons conducted the movements of sleepers was likewise part of English culture in the seventeenth century. In Thomas Killigrew’s comedy *Thomaso, or the Wanderer* (1654) one of the characters, on seeing his lover pace up and down the room, observes: “There is no danger in questioning this Noctambule; I have heard of them would walk upon Ridges of houses, guided by the Fiend, without danger, unless you awake them”.^40^ There is also an intriguing set of candle marks dating from the second half of the seventeenth century that was discovered on the ceiling of a bedchamber in a house in Suffolk. Timothy Easton and Sasha Handley have speculated that these may have been placed there as a counter-diabolical measure to prevent the occupant from walking in her sleep. The marks follow a trail along the ceiling from the bedstead down the stairs to the kitchen.^41^

The notion that self-destructive behaviour was instigated by the devil was widely accepted at this time, as was the concept that demonic beings could stir up humours in the body and deceive the dreamer with *phantasma*.^42^ It is therefore unsurprising that the devil might be supposed to play some role in cases where sleepers endangered themselves. Whether demons played a role in physically transporting the body, asleep or awake, walking on rooftops or flying through the air, was a more controversial matter.^43^ In Protestant England Kramer’s theory that calling a *noctambulo*’s name acted as a form of dispossession was particularly problematic. By the late sixteenth century the questions raised by the *Malleus* concerning rebaptism had become part of an international confessional conflict over whether the rites of the Catholic church were effective in curing demoniacs. In *The Discovery of Witchcraft* (1584) Reginald Scot took the Reformed position, which did not accept the efficacy of the rite of exorcism contained in the Roman Catholic service of baptism. This had been expunged from the English Book of Common Prayer in 1552. He therefore dismissed the notion that people walked in their sleep because they had not been baptised properly (and therefore could be cured by calling out their name) as “beggarly, foolish and frivolous”.^44^

Lorraine Daston and Katharine Park have identified the second half of the sixteenth century as a period in Europe when physicians sought to demystify marvels, explaining them in terms familiar to natural philosophy.^45^ Sometimes they appealed to the notion that there were hidden powers in nature itself that were not yet understood, but could cause marvellous effects without the aid of personal spirits. Scot subscribed to the view that there were occult virtues in various stones and herbs that could cause people to reveal secrets in their sleep.^46^ This was the position of Cornelius Agrippa, who had written that the effects of fumigations and ointments on the skin could cause people to both talk and walk in their sleep.^47^

However, the writer who had the greatest influence on how the *noctambuli* were depicted in English medicine was the Dutch physician Levinus Lemnius, whose explanation for the skills of the *noctambuli* was based entirely on humoral theory. He gave a detailed analysis of the somatic causes behind the *noctambuli*’s amazing feats in a chapter entitled “Of those that come forth of their Beds, and walk in their sleep, and go over tops of Towrs, and roofs of houses, and do many things in their sleep, which men that are awake can hardly do by the greatest care and industry”, in *Occulta naturae miracula* (1559) (“The Secrets of Nature’s Miracles”). In this highly influential work, Lemnius argued that the human body contained hidden potentials that could explain a whole range of phenomena that could appear supernatural or miraculous: the power of a mother’s imagination to shape a baby in the womb; the natural causes of epilepsy, ascribed by the common people to the work of saints; the ability of some mad or frantic people to speak in strange languages, simulating demonic possession. The chapter on the *noctambuli* was clearly addressing the belief that sleepers were led by demons. This unusual behaviour of the *noctambuli* was, he claimed, entirely natural, and was most common in young people, whose spirits were active and minds hot. Their bodies were light and full of frothing blood, making them quick and agile, and the spirituous vapours rose up into their heads, enabling them to remain balanced at great heights. He likened people of this constitution to buoys bobbing about on the sea, which remained anchored and did not sink to the bottom because they were filled with gas. The *noctambuli* showed no fear while they climbed because sleepers, like the drunk or mad, had no sense of their surroundings. However, if they were awakened by someone calling out their name this would have such a dramatic effect upon the body that they would fall:

When they do these things if you speak to them in a known voice, or call them by their christian names, they will fall being frighted thus; their spirits being dissipated, and their natural force discussed whereby they perform these things.

It was therefore dangerous to call out to a *noctambulo*, but there was no inherent power in the Christian name itself. Lemnius advised that it was best not to disturb them, but to let them return to bed of their own accord where they would wake up safely, remembering nothing of the episode.

Lemnius also explained what would happen in people who were moved by dreams, but whose complexions were not so hot. In this case, the spirits would not be violently enough stirred up to lift the body, so the sleeper would “onely cry out and leap a little”, but stay in their bed.^48^

*Occulta naturae* did not become available in English until 1658, when it was published as *The Secret Miracles of Nature*. However, its influence on English medicine can be seen much earlier. Thomas Hill in his treatise on dreams, published in 1576, explained that whether or not people who moved in their sleep would get out of bed depended on the strength or weakness of the vigorous spirits.^49^ James Hart’s description was based on “a late writer” (clearly Lemnius) who attributed the phenomenon to “hot and vaporous spirits, arising from a commotion and heat of the blood”. He wrote that these people ventured fearlessly upon rooftops, narrow beams and bridges because, the faculty of common sense being at rest, they did not discern the danger:

Unlesse by loud houping and crying the party be awakened out of sleep. If they be suddenly awaked, then are they in danger of sudden precipitation, of falling downe head long, all the spirits and powers of the body then leaving the extreme parts hands and feet, and flying to succour the feeble heart now assaulted with no small feare.^50^

Similarly, Thomas Tryon’s account of the *noctambuli* was also based on Lemnius’ argument that it was the bodily complexion of the sleeper that made it possible for them to rise up in their sleep. He described those who were able to perform amazing feats during sleep as:

*young people*, in the flower of their years, and strength, of *Sanguine Complections*, active, sprightly, and full of Blood … of a thin and eurious contexture, small bulk, but of great agility and a fervent mind, whence, if they can but take hold of any thing with their Fingers and Toes, being then void of all fear, because insensible of any danger, they sustain themselves thereupon, and accomplish such things, as waking they would dread to attempt.

People whose bodily humours were less hot and agitated would cry out and fling themselves about in the bed, but would not be able to get out. Tryon suggested that people given to walking in their sleep could prevent episodes by eating a spare diet, endeavouring to keep their minds in a cool temper and, in some cases, the use of blood-letting.^51^

In his study of medical accounts of the incubus disease in the fifteenth century, Maaike van der Lugt found that medical writers at this time were influenced by debates in demonology. Prior to this time descriptions of the incubus disease did not contain discussions of the incubus demon, which was a sexual being in many ways quite different from the crushing spirit to which the sleeper’s terrifying experiences were attributed in European popular culture. Discussions of the incubus demon were absorbed into medical texts as physicians sought to find somatic explanations for the supernatural phenomena described in demonological discourse.^52^ A similar process of assimilation can be observed in accounts of the *noctambuli* in the following centuries. Kramer’s descriptions of the demoniacal sleeper led over rooftops by dark spirits influenced how Lemnius described the phenomenon in the sixteenth century, which then formed the basis of English accounts in the following decades.

### Spectres and imagined travels

English depictions of the *noctambuli* were also influenced by continental debates over the imagination in which the behaviour of sleepers was presented as evidence of the kinds of sensory delusions that cast doubt on accounts of ghost-sightings and nocturnal travels. The notion that the imagination could become out of control during sleep, leading to unwanted behaviour, was not new in the sixteenth or seventeenth centuries. As MacLehose has shown, this was a problem that had preoccupied natural philosophers and theologians as early as the thirteenth century.^53^ However, in the early modern period, the context in which this was discussed shifted. The focus was no longer on the moral problems of the sleeping imagination, and how sleepers might cause injury to themselves or others, but on what such sensory delusions suggested about the existence, or otherwise, of a supernatural realm. The widely shared perception that those who walked in their sleep were guided by false images provided evidence that sleepers were particularly vulnerable to such stimuli, which was central to the sceptical position that regarded visions of spectres and sabbatical travels as the products of the delusions of madness or dreaming.^54^

In *A Treatise of Specters or Straunge Sights*, which appeared in English in 1605, Pierre le Loyer included stories of the *noctambuli* as evidence of the power of sensory delusions created by varying conditions of the body. Just as a person with a fever imagined everything they touched to be hot, so a person asleep moved entirely by the sensation of dreams, unaware of the body’s external surroundings.^55^ Le Loyer was a likely source for undoubtedly the most famous depiction of sleepwalking in English literature – the scene from *Macbeth* (first performed in 1606) in which Lady Macbeth is observed by her lady-in-waiting and a physician to walk in her sleep. This dramatic depiction of a *noctambulo*, alongside the bearded women, represented to the audience wonders that medicine had sought to demystify through reference to the effects of hot humours or the powers of the imagination upon the body.^56^ Connected to this was the theme of vision affected by corrupted imagination. As has been amply demonstrated by Stuart Clark and Suparna Roychoudhury, many of the sights in the play can be interpreted as *phantasma* of a melancholic imagination or tricks of the eye – the vanishing women, the dagger, the ghost, the moving forest, the procession of kings conjured up by the witches – the characters continue to question whether these are true apparitions or products of their own troubled state of mind.^57^ The sleepwalking scene extends this theme of sensual delusion. The physician remarks that the Lady’s eyes are open; “Ay, but their sense is shut”, the gentlewoman replies. When later in the play he is asked to give an account of Lady Macbeth’s condition his explanation is that “she is troubled with thick-coming fancies/That keep her from her rest”.^58^

The *noctabmuli* were also included in support of the argument that sabbatical travels were the product of fantastical dreams, rather than the actual power of demons within the physical world. In this regard, accounts of the *noctambuli* were similar to tales of women who were observed to act out the motions of flight while under the influence of powerful drugs – they provided physical evidence of how the imagination behaved during periods of unconsciousness.^59^ Hieronymus Nymann gave a highly influential series of lectures on the imagination at the University of Wittenberg in 1593, which addressed this subject. Here he argued that the powerful illusions produced by the imagination when under the influence of madness, melancholy or the vapours of sleep were responsible for a range of supposedly supernatural phenomena, including attacks of the incubus demon, werewolves and aerial travels. The figure of the *noctambulo*, who climbed to great heights acting entirely by the guidance of the imaginative faculty, demonstrated the complete subordination of the senses to the imagination during sleep.^60^

Robert Burton included a typically potted version of Nymann’s arguments in a section on “force of the imagination” in *The Anatomy of Melancholy* (1621). The *noctambuli* offered tangible proof of the powerful effects of the melancholic vapours of sleep, which were responsible for “all those tales of Witches progresses, dancing, riding, transformation, operations, &c”.^61^

### Disease or bewitchment?

While the influence of debates over transvection can clearly be seen, English writers in medicine and natural philosophy were less preoccupied than continental writers with the question of transportation, which is not a feature of English witchcraft trials. Their main concern was how a *noctambulo* might be mistaken for a spectre, or for a person suffering the effects of *maleficium*. Concerning the first, Ludwig Lavater had made the connection in *Of Ghostes and Spirites Walking by Nyght*, which was published in English in 1572.

There be many which haue suche a kinde of disease, that they walke in their sléepe: … who in theyr sléepe, climed up to the top of the house. …. If a man sée suche a one walking in the night, eyther apparled or naked, and after heare him say he was at y^e^ same time in his bed, he will straight thinke, it was his soule that he sawe, the like will he do if he heare suche a one at his owne house.^62^

The *noctambuli* were seen as eerie figures whose abilities and behaviour mimicked those of disembodied spirits or the possessed. This was expressed most clearly by Thomas Willis, a hundred years after Lavater, in a passage on sleep which made a number of observations about the apparently demonic nature of disturbed sleep patterns and dreams:

I knew a certain Man, who was wont after this manner to walk a-Nights like a Spectre … Yea, it is observed of most of these Night-walkers like Spirits, that being awakned, they scarce remember any thing of what they did, or acted in their Sleep; as if they suffer’d something that was different from other Dreamers.^63^

As a result of their uncanny nature, there was a tendency in the seventeenth century to frame somatic explanations for the behaviour of the *noctambuli* as a materialist alternative to demonological causes. An example of this can be found in Edward Jorden’s *A Briefe Discourse of a Disease Called the Suffocation of the Mother* (1603). Jorden’s role in promoting scepticism around English cases of possession is well established, as is the importance of epilepsy, hysteria and melancholy across Western Europe and America in explaining the fits and hallucinations of the supposedly possessed.^64^ What is not so well known is that one of the symptoms Jorden described in his essay was unusual forms of sleep. There were similarities in Hippocratic medicine between the state of sleep and madness: both were a deprivation of sense with a physiological cause, the difference being that in (normal) sleep motion was also disabled. Jorden’s discussion of diseased forms of sleep therefore followed on naturally from his description of the symptoms of melancholia as both were diseases in which a person became subject either to false memories, images or judgements, or in which the senses became abolished altogether. This occurred in sleeping diseases, which included coma vigil, lethargy, apoplexy, and also dreams “where sometimes besides the depravation of the fantasie they wil walke, talke, laugh, crye, &c”.^65^

John Cotta included a much lengthier description of how the movements of the *noctambuli* resembled bewitchment in a chapter on wonderful diseases in his treatise against empirics. As in Jorden’s essay, the actions of the sleeper were seen as a continuation of a derangement of the mind and senses that manifested itself in both waking and sleeping. His conclusions were based on his experiences with a distinguished family in Warwickshire whose daughter he cured of a strange illness after a number of other physicians had failed. His record is a rare example of a first-hand account of an actual case of sleepwalking from England in this period. He describes how the girl, who was almost thirteen, was subject to fits of violent shaking and at other times would lie in a death-like trance. This situation continued for three weeks:

While these fits at any time discontinued, she either slept, or (at least all her outward senses slumbring) her imagination still led her hands unto many and divers continuall actions and motives, which argued in their folly great fatuitie and defect of reason and understanding, yet manifested the business and depraved motion of her oppressed imagination, which therefore continually imployed her fingers to imitate many usuall exercises of her health (as dressing and attiring the heads of such women as came neare unto her). In all these actions and motions she neither had nor used the helpe of any other sense but onely the feeling with her hand, whereof she seemed also altogether deprived in all other things, except onely those whereto her imagination (which is mistresse and great commander of all the senses) lead her feeling.

Cotta went on to describe how they tried to wake her with pinching, but she was unresponsive. The case proved to be natural, however, which was shown when Cotta’s medicines successfully restored her understanding.^66^

The distinctions Cotta drew between natural disease and the symptoms of *maleficium* were highly influential in England and America. The puritan clergyman Richard Bernard included the Warwickshire case in a chapter on “Strange diseases … from natural causes” (which was basically an abridged version of Cotta) in *A Guide to Grand-Jury Men*. First published in 1627, this remained an important document throughout the seventeenth century in both Britain and America, which was consulted by the magistrates involved in the infamous witchcraft trials that took place at Salem.^67^ The purpose of the works of Cotta and Bernard was not to disprove the existence of witchcraft, but to educate physicians and those involved in the legal process in how to discern the differences between natural and unnatural illnesses. Their explanations therefore did not rule out the existence of a supernatural realm, but they did curtail, to some extent, the situations in which supernatural agents were supposed to operate.^68^

### The good angel

Despite the importance of humoral explanations for the *noctambuli*, supernatural – or, rather, natural magical – explanations were entertained in the second half of the seventeenth century. This was to a large extent owing to a surge of interest in Paracelsian medicine and natural magic, which rejected the notion that the natural world could be explained entirely in materialist terms, and the publication in the 1650s of a number of works that took a mystical approach to states of unconsciousness and dreaming. As a result, the causes of people moving about in their sleep became part of a wider debate over the existence of invisible forces of sympathy and antipathy, unconscious “intelligence” and prophetic dreaming.

An enquiry to *The Athenian* in the 1690s told the story of a young woman from Windsor who had dreamt that her father was killing her mother. She had run all the way to her aunt’s house in her sleep, opening several doors, and in her bare feet. The enquirer asked for information as to how this was possible. The editor (either Dunton himself, or one of his co-editors), after giving some unremarkable explanations in terms of the effects of melancholic dreaming on bilious constitutions, then added:

Some Persons do believe, that *Sleep-walkers* are actuated by some *good or bad Spirit*, which get into the Body as into a Ship whose Pilot is asleep, and guides it at Pleasure, carrying it any where, and returning it to the same place again: And to strengthen this Opinion, they tell us of one *Levinus Lemnius*, who walked with his Feet against the Rafters, with his Head downward, and yet *fast asleep*.^69^

Lemnius himself had never made any such claim. The editor based his response on a public lecture given in the 1630s at the *Bureau d’Adresse* in Paris, a forum for the discussion of cutting-edge topics of the day in natural philosophy, such as magnetism, alchemy, contagion and witchcraft. A collection of the lectures were published in English in 1664. The speaker proffered that the enhanced abilities of the *noctambuli* could not be accountable to somatic causes alone:

but to some spirit good or bad, whether such as they call aerial Hob-goblins, or others; which insinuating into the body, as into a ship whose Pilot is asleep, governs and guides it at pleasure; and as a thing abandon’d to the first occupant, carries it where it list, and then returns it to its former place.

This, he argued, was the only way to explain the extraordinary feats of sleepwalkers recorded in wonder books, such as the man who walked upside down on the ceiling (a story he attributed erroneously to Lemnius).^70^

The editor of the Athenian rejected the idea that sleepwalking was caused by anything other than bodily processes. However, this is only one example of the interest in good angels or intelligences exerting an influence upon sleepers. By the second half of the century the *noctambuli* had become embroiled in debates over prophetic dreams and the state of ecstasy. This shift in context can be seen in Meric Casaubon’s *Treatise Concerning Enthusiasme* (1655), which contained a short passage on the *noctambuli* in a chapter on divination. Those who have knowledge of the secrets of the body, he argued, will be able to see that there are many instances in nature that the “vulgar” might mistake for supernatural gifts of prophecy. These might include a heightened sense of smell, or particularly wonderful powers of memory. He also gave the example of people walking in their sleep as a phenomenon “that might be thought to have some affinity with possession *and Enthusiasme*”, [*my emphasis*].^71^

In the final part of the same chapter, Casaubon considered the possibility that divination might be natural to the human subject. This theory was based on a number of ideas, such as the existence of an *intellectus agens* (active intelligence) that was separate from the soul or rational faculty, or the sensitivity of the sick, dying, those in trances or sleeping persons to emanations of species. Though not entirely dismissive of these notions, he thought many of them too abstruse and, on balance, preferred the insights of a rational mind.^72^ Others, however, took a keen interest in such subjects. In the seventeenth century, mystical concepts of a natural but immaterial power within the body – such as a genius or astral body, or a magnetical element contained within the blood or spirits – provided both a defence for belief in prophetic dreaming and an alternative explanation for the abilities of the *noctambuli*.

The most radical expressions of this alternative stream of mystical thought can be found in the works of continental writers. An example is Cornelius Agrippa’s *De occulta philosophia*, published as *Three Books of Occult Philosophy* in 1651. Agrippa believed in levitation and wrote at length about the powers of the imagination. He described how the Chaldeans discovered that, through ascetical practices and by fixing the mind on God, it was possible for the body to become filled with light, shining like the stars. On extraordinary occasions, this luminesce energy could be so powerful that the body would rise “as straw lifted up by the flame of fire”. The *noctambuli*, he argued, provided evidence that levitation was possible:

He may the lesse wonder at this, who hath seen those famous melancholick men, who walk in their sleepe and passe through places even unpassable, and ascend even unaccessible places, and exercise the works of those that are awake, which they themselves being awake could not do; of the which things there is no other reason in nature, than a strong and exalted imagination: but this power is in every man, & it is in the soul of man from the root of his Creation.^73^

Belief in levitation had its origins in Pythagorean philosophy and the notion of the astral body. According to this tradition the soul pre-existed the body and descended through the heavens in order to join it. Passing through the stars and spheres, it acquired a sidereal encrustation, forming the astral body. This ethereal body became imprisoned within the material body, along with the soul, which longed to return to the heavens. Through various ascetic practises and rituals of purification, the body could become light and dry so that the astral body, returning to its lustrous quality, would be able to ascend, carrying the material body with it. Generally, Renaissance writers avoided an overt endorsement of the notion of the astral body because of its associations with the unorthodox doctrines of the pre-existence of the soul and metempsychosis (reincarnation).^74^ However, versions of this belief system, which conceptualised the body in a very different way from orthodox medicine, became popular in esoteric circles from the late sixteenth century and can be seen to influence ideas about ecstasy, prophecy and the *noctambuli* in English texts from the mid-seventeenth century.^75^

An example of this is in the work of the clergyman philosopher Alexander Ross *Arcana Microcosmi, or The Hid Secrets of Man’s Body*, published in 1652. He gives an expansive account of the usual physiological reasons advanced for the behaviour of the *noctambuli*, but then appends this one comment – that they do not hurt themselves during their dangerous exploits because “their Genius or good Angel is carefull of them”.^76^ In Paracelsian medicine “genius” was another term for an astral body. This was explained in the works of Oswald Croll:

The other part therefore of Man, or this sydereall body is called the Genius of man, because it proceedeth from the Firmament; it is called *Penates*, because it is in our power and born with us, the shadow of the visible body, *Lar domesticus*, the good or bad household or private Angell, the Umbratile or shadowy Man, the familiar Homuncle (or little Man) … It is also called the imagination, which incloseth all the Astra’s, and is indeed all the Astra’s or Starrs and holdeth the same course, Nature and power with heaven.^77^

Ross’s inclusion of this mystical idea in an otherwise orthodox account of the causes of people rising in their sleep undermines the sceptical position that saw the *noctambuli* purely in terms of diseased imagination. It acts like a loose thread in the text, unravelling the certainty established in the previous passage that humoral theory could fully explain the abilities of the *noctabambuli*.

It is also clear from unpublished correspondence that those in intellectual circles who sought to find evidence for the existence of the supernatural realm were reading texts which interpreted the behaviour of the *noctambuli* through the framework of mysticism. In June 1657 the natural philosopher and clergyman John Beale, a firm believer in prophetic dreaming, wrote a letter to Samuel Hartlib giving his opinion on various writers on the subject that Hartlib had suggested he read. These included Casaubon alongside more esoteric writers. Beale was such an avid reader and writer on dream theory, sleep and altered states of consciousness that at one point he could not send Hartlib a copy of the latest chapter of his own treatise on dreams because it was buried under a pile of paper. He was influenced by Paracelsian concepts of sleep and dreaming, such as the notion that the knowledge of powerful cures could be received through dreams.^78^ He began this particular letter by expressing his disapproval of the “disputants & infidells” who ascribed all dreams to chance, before turning to explanations of “how the soul spirite [*altered*] in dreames, & fits of bodily weaknes, peepes through the loopholes of our earthly prison into the region of Eternity”. Here he wrote with qualified approval of the work of the Flemish physician Jan Baptiste Van Helmont.^79^

Van Helmont’s views on dreaming could be found in his controversial treatise on *The Magnetick Cure of Wounds*. This was first written in 1621, but it became more widely available in 1650 when an English version was published by Walter Charleton in response to the weapon-salve controversy. The weapon-salve, and Sir Kenelm Digby’s version of it, has been written about extensively elsewhere. Briefly, the debate focused on how far explanations based on the natural powers of sympathy and antipathy could be stretched in defence of a wonder-cure that looked suspiciously like witchcraft.^80^ Helmont’s treatise ranged far beyond the cure itself, addressing questions about prophecy, sleep, aerial travels, astral projections (“ghosts”) and the bleeding of corpses, all of which, he argued, were natural phenomena attributable to vital powers in the blood or a separate intelligence.

Helmont subscribed to a Judeo-Christian version of mysticism which explained the tripartite division of the human person (soul, astral spirit and body) through the Genesis narrative of the Fall. On account of eating the forbidden fruit, Adam became encased in a corrupting and debilitating material body, but some of the powers of the immaterial prelapsarian body were retained within the blood and the semi-corporeal spirit.^81^ His Behemenist Christianity led him to a radical rejection of the prevailing medical model of sleep. The first occurrence of sleep recorded in scripture is when God placed Adam in a deep sleep in order to remove the rib from which he formed Eve. The fact that this took place before the Fall was, for Van Helmont, of primary significance:

The Schools indeed teach, that Sleep is caused by vapours lifted up out of the Stomack into the Brain, stopping or intercepting the passages of the Senses, Motion, Speech, Judgement, &c. which things surely, I being as yet a young man, judged to be ridiculous: For in very deed, so a disease had been before sin; because sleep should be a disease; to wit, there had been a flatulent a vapoury *Palsie*, and Temporary madness, both in a body then as yet, not capable of suffering, and a life immortal.^82^

Following this logic, Van Helmont did not see the movements of sleepers as evidence of disease or incapacity, but rather as proof that superior powers of the body had been awakened. During sleep, when the sensual awareness of the material body (which Helmont refers to as “the science [knowledge] of the apple”) is temporarily suspended, mankind regains something of his original capacity:

But in regard ever now and then the science of the aple is suspended and chained up in the leaden fetters of sleep: hence it is also, that sometimes our dreams are propheticall … for when the *interior magick* of the *Soule* stands unmolested and free from any disturbance of the *Science* of the *inderdicted fruit*, then and onely then doth the *intelligence* keep holy-day, enjoy an halcyon Calme, and freely diffuse it selfe through all its royaltie: for thus doth it, when it demergeth it self into the inferiour and subordinate faculties, safely conduct and lead along those that *walk in their sleep*, over such horrid praecipices, where the strongest brained man awake durst not adventure to clime.^83^

Writing about Van Helmont in a different context Beale found him “fuller of lofty words, then of wonderfull deedes”.^84^ His own claims about the powers of sleepers were rather more modest, but were based on first-hand observation. In his letters and his unpublished treatise on dreams, which he sent to Hartlib, he wrote at length about the susceptibility of the sick to the reception of divinatory or premonitory dreams, which he saw as “manifest proofe of the divine nature of Mans spirite”. These revelations came during sleep when the senses were locked up, and sometimes revealed themselves in dramatic gestures. He gave the example of a young man who had been his chamber fellow – “a retyred melancholy youth” – who “in his first sleepe frequently starting out of it, & beating the bolster with an enraged outcrye; This youth did duely give us some days præadvertisement of the death of his neere friends”.^85^

## Conclusion

Sasha Handley, in her article on sleepwalking in the long eighteenth century, records how members of the Romantic movement experimented with a new form of artificially induced sleepwalking that emerged in the 1780s – magnetical somnambulism. The poet Percy Shelley asked his wife to magnetise him in order to connect with his creative genius, but she had to discontinue the practice when he tried to climb out of a window.^86^ George Pigman III in his recent history of dreaming also detects a rise of interest in the special abilities of sleepers during the eighteenth century, which were observed in sleepwalking, problem-solving, creativity and powers of memory.^87^ This represented a significant change in how sleepwalking was perceived over an 150-year period. Whereas, in the early decades of the seventeenth century, sleepwalking was associated with diabolical instigation and delusional madness, by the end of the eighteenth century it had become a desirable state in avant-garde circles that was actively sought out.

This article goes some way to explaining how this shift took place. The associations between sleepwalking and genius began in the mid-seventeenth century when intellectuals in search of evidence for prophetic dreaming turned to alternative conceptions of the sleeping body found in the traditions of hermeticism and Paracelsian medicine. Van Helmont’s theory of animate powers within the blood offered a mystical explanation for the extraordinary powers of the *noctambuli*, as described in wonder books and witchcraft treatises, and suggested that prophetic dreams were natural. Paracelsian and Helmontian theories of the imagination have been seen as foundational for the later movement of animal magnetism.^88^

This development corroborates the findings of Craig Koslofsky, who argues that, during the seventeenth century, broad cultural shifts were taking place across Europe that led to an embracement of the darkness of night as a positive presence that offered opportunities for unity with the divine.^89^ The Behemenist views of night that he writes about were not only affecting religious cultures, but were also influencing developments in medicine and science. Whereas in the late medieval period Kramer had regarded the movements of sleepers as evidence of malevolent forces at work in the dark of night, these seventeenth-century writers saw them in terms of the work of benign intelligences or forces of sympathy and attraction.

Michael Hunter in his study of investigations of the phenomenon of second sight (closely related to premonitory dreaming) has found a similar pattern. Interest in second sight was instigated by a desire to prove the existence of a realm beyond the material. In the next century, the concept became part of the literature of the Romantic movement.^90^ This study of the *noctambuli* provides further insight into the ambivalent legacy of the fraught debates of the second half of the seventeenth century, identified by Hunter, and the role that medicine played in those disagreements. The shift that took place was not from a supernatural view of sleep towards a material one (the orthodox medical explanation for sleepwalking remained largely unchanged from the medieval period), but from a pessimistic view of night as filled with dark spirits towards a more positive view of the forces at work within the natural world and the body. For a thinker such as Beale, Helmont’s Behemenist views of creation were an attractive alternative to the atheist materialism represented by the Hippocratic school.

There is a tendency to assume that medicine was a foil for supernatural beliefs, with physicians presenting materialist explanations for strange behaviour of the body, but belief in supernatural causes continuing in popular culture. It is easy to find evidence to support this assumption because of the very materialist nature of humoral medicine. Such was the explanatory power of this model that it was able to account not only for vivid dreams, but also for superhuman actions accomplished by sleepers, which were attributed entirely to the effects of imagination and aerial spirits. The fact that figures as distinct in their approach to dreaming as Thomas Tryon and Robert Burton explained sleepwalking in these terms is testament to the widespread acceptance that disturbances within the bodily humours could produce astonishing effects. Nevertheless, viewing medicine and the supernatural realm in terms of a dichotomic struggle can also be misleading because it obscures the diversity and debate within medicine itself, and side-steps the question of what constituted “natural” explanation. While humoral theory remained transcendent well into the eighteenth century, its domination was never complete. This was especially true in intellectual circles where interest in views of the body and the cosmos deriving from esoteric traditions, and dissatisfaction with scholasticism, was an important driving force behind the reform of medicine and science that took place in the second half of the century. The same Paracelsian body of thought that lay behind the weapon-salve controversy and inspired early experiments in chemistry, also supplied counter views of the sleeping body that sowed the seeds of interest in the paranormal in later centuries. Sleep studies, therefore, do not always support the disenchantment theory.

## Notes

1. Dunton, *Dublin Scuffle*, 354-[356] (misnumbered as 340).

2. MacLehose, “Sleepwalking, Violence and Desire”; Handley, “Sleepwalking, Subjectivity”; Idem., *Sleep in Early Modern England*, ch. 6. Handley develops her thesis in a recent article showing how sleepwalking was used in conduct books to demonstrate the dangers of immoderate passions: Idem., “Deformities of Nature”.

3. MacLehose, “Sleepwalking, Violence and Desire”, 612–617.

4. Handley, *Sleep in Early Modern England*, 188–196.

5. Hunter, *The Decline of Magic*; see also Daston and Park, *Wonders and the Order of Nature*; Daston, “Preternatural Philosophy”; and Clark, *Thinking with Demons*.

6. Thomas, *Religion and the Decline of Magic*, 151–153.

7. Mack, *Visionary Women*; Crawford, “Women’s Dreams in Early Modern England”; Plane and Tuttle (eds.), *Dreams, Dreamers, and Visions*; Levin, *Dreaming the English Renaissance*; Hodgkin, O’Callaghan and Wiseman, *Reading the Early Modern Dream*; and Plane, *Dreams and the Invisible World*. Nashe, *The Terrors of the Night*, sig. Ciii(v).

8. Following the tradition established by Carlo Ginzburg in the 1960s, Emma Wilby and Julian Goodare use insights from cultural anthropology to suggest a vital role for British pre-Christian shamanistic cults in generating narratives of nocturnal travels: Ginzburg, *Night Battles*; Wilby, *Cunning Folk*; Goodare, “Cult of the Seely Wights”. Edward Bever draws on the latest insights from modern science concerning out-of-body experiences and dream states in children and the elderly in order to advance the thesis that dreams were the origin of many witchcraft confessions: Bever, *Realities of Witchcraft*, 93–150.

9. Davies, “The Nightmare Experience”. See also Oats, “Cheese Gives you Nightmares”.

10. Janine Rivière, *Dreams in Early Modern England*, ch. 4. See also idem., “Demons of Desire”.

11. Tryon, *A Treatise of Dreams & Visions*, 21–22.

12. The earliest use I have found is in an English translation, published in 1664, of a collection of lectures on natural philosophy given at the *Bureau d’Adresse* in Paris (examined later in this article). The translator renders the passage as “Sleep-walkers, call’d by the Greek *Hypnobatae*, are such, as, rising out of their beds in the night, walk about in their sleep, and do the same things as if they were awake” (Bureau d’Adresse, *General Collection*, 197). The Greek is a combination of *ϋπνος* (sleep) and *βατης* (walker). There is also an early use of the Latin form of sleepwalkers – “*somnambuli*” – in John Bulwer’s 1649 treatise on mind and motion, *Pythomyotamia, or, A Dissection*, 34. Nathaniel Wanley, writing in the 1670s, used the term “somnambuloes”, Wanley, *Wonders of the Little World*, 592.

13. Handley, “Sleepwalking, Subjectivity”, 307–308.

14. Griffiths, “Meanings of Nightwalking”.

15. Scot, *Discovery*, 103.

16. Ekirch, *Day’s Close*, 59–84 and 261–262; Griffiths, *Lost Londons*, 332–342. Not all movement at night was proscribed, but the activities that were considered legitimate by night and by day differed, therefore the sleepwalker’s performance of daily activities could be seen as transgressive. The broad category of “nightwalker”, which did not distinguish between malevolent human and supernatural agents, has a long history in the English language. Anglo-Saxon medicine contains remedies against attacks of “the devil, elves and nightwalkers”: Meaney, “The Anlgo-Saxon View”, 20–21.

17. American Psychiatric Association, *Sleep-Wake Disorders*, 46–50; The American Academy of Sleep Medicine provides a lay-persons description of sleepwalking on their website *sleepeducation.org*, at http://sleepeducation.org/sleep-disorders-by-category/parasomnias/sleepwalking/overview-facts [accessed 18/10/2020].

18. Daston and Park, *Wonders and the Order of Nature*, 198; Walsham, “Miracles in Post-Reformation England”, esp. 304–306; on the popularity of printed tales of prodigies and wonders in Protestant England see Crawford, *Marvelous Protestanitsm*; on the development of the Protestant wonder book genre see Soergel, *Miracles and the Protestant Imagination.*

19. Wanley, *Wonders of the Little World*, 12–13, 592–594.

20. Goulart, *Admirable and Memorable Histories*, 199–204 [mis-numbered 188]; originally published as *Thrésor d’histoires admirables et memorables de nostre temps* (Paris, 1600). On Goulart’s reception in England see Kay, “Who Says ‘Miracles are Past?’”

21. Wanley, *Wonders of the Little World*, 592–594; and Sandys, *Anglorum Speculum*, 447.

22. Lupton, *A Thousand Notable Things*, 34–35.

23. Le Loyer, *A Treatise of Specters*, 52; and Ross, *Arcana microcosmi*, 133.

24. Wanley, *Wonders of the Little World*, 592–593.

25. Lupton, *A Thousand Notable Things*, 34–35.

26. Pierre Le Loyer, *A Treatise of Specters*, 52; Goulart, *Admirable and Memorable Histories*, 199–204 [mis-numbered 188]; and Wanley, *The Wonders of the Little World*, 592–294.

27. Goulart, *Admirable and Memorable Histories*, 200, 204 [mis-numbered 188].

28. Goulart, *Admirable and Memorable Histories*, 199, 202.

29. Goulart, *Admirable and Memorable Histories*, sig. a(v); and Lupton, *A Thousand Notable Things*, 35.

30. Jonson, *The Magnetic Lady*, 203 (Act 5, Sc. 8: 11–16).

31. Goulart, *Admirable and Memorable Histories*, 202–203.

32. Hart, *Diet of the Diseased*, 340–341.

33. On the physiology of sleep see Handley, *Sleep in Early Modern England*, 22–23.

34. Hart, *Diet of the Diseased*, 338–340.

35. Gowland, “Melancholy, Imagination, and Dreaming”, 66–67.

36. In “On Sleep” Aristotle wrote that “Some persons move in their sleep, and perform acts like waking acts but not without an image or an exercise of sense-perception; for a dream is in a certain way a sense-impression”.: Aristotle, *Complete Works of Aristotle*, i.725.

37. More, *A Dialoge [sic] of Comfort*, sig. kii^v^-kiii^r^.

38. De Torquemada, *Spanish Mandevile*, 89–90; and Goulart, *Admirable and Memorable Histories*, 202–203.

39. Mackay (ed.), *Hammer of Witches*, 295–296, 457. Alain Boureau has suggested that the association between sleepwalking and witches’ transportations began when women were found waist-deep in water in the morning and did not know how they had got there. It was believed they fell from the sky when their Christian name was called: Boureau, *Satan the Heretic*, 152–156.

40. Killigrew, *Comedies and Tragedies*, 428 (*Thomaso* Act 4, Sc. 1).

41. Handley, *Sleep in Early Modern England*, 101; and Easton, “Candle Powers”, 58.

42. MacDonald and Murphy, *Sleepless Souls*, 5–7; and Hunter, “‘Between the Bridge and the Brook’”, 249–250. On demonic *phantasma* see Clark, *Vanities of the Eye*, 125.

43. On the metaphysics of transportation and the debate between sceptics and the inquisitors see Clark, *Thinking with Demons*, 185–186, 191–192.

44. Scot, *Discovery*, 267–268. On the rite of exorcism in baptism see Coster, *Baptism and Spiritual Kinship*, 69–70; Rosen, *Witchcraft in England*, 227–229; and Thomas, *Religion and the Decline of Magic*, 570–572. For the continental dimension see Nischan, “The Exorcism Controversy”, esp. 47–48. On confessional propaganda and conflicts over dispossession see Sluhovsky, “A Divine Apparition or Demonic Possession?”; and Levack, *The Devil Within*, 85–94.

45. Daston and Park, *Wonders and the Order of Nature*, 159–172.

46. Scot, *Discovery*, 132–133.

47. Agrippa, *Three Books of Occult Philosophy*, 91.

48. English translation taken from Lemnius, *Secret Miracles*, 99–101; the original can be found at Lemnii, *Occulta naturae miracula*, 113–115.

49. Hill, *Moste Pleasuante Arte*, Ciiii^v^-Civ^r^.

50. Hart, *Diet of the Diseased*, 340–341.

51. Tryon, *Treatise of Dreams & Visions*, 21–24.

52. Van der Lugt, “Incubus in Scholastic Debate”.

53. MacLehose, “Sleepwalking, Violence and Desire”; and idem, “Captivating Thoughts”.

54. Clark, *Vanities of the Eye*, 204–235, 309–322.

55. Le Loyer, *Treatise of Specters*, 52. Originally published as *IIII livres des spectres ou apparitions et visions d’esprits, anges et demons se monstrans sensiblement aux hommes*. Angers, 1586.

56. Hirsch, “‘What are These Faces?’”

57. Clark, *Vanities of the Eye*, 236–265; Roychoudhury, “Melancholy, Ecstasy, Phantasma”, 217–219.

58. Shakespeare, *Macbeth*, 272 (Act 5, Sc. 1: 24–25), 281–282 (Act 5, Sc. 3: 38–39). On the significance of Le Loyer’s treatise in relation to *Macbeth* see Clark, *Vanities of the Eye*, 246–247.

59. Broedel, *The Malleus Maleficarum*, 112–113. For an overview of the literature see Beecher. “Witches, the Possessed”.

60. Nymann, *Oratio de imaginatione*, the section on sleep is at 56–59, sleepwalking at 57. On Nymann’s lectures and their reception in Europe see Clark, *Vanities of the Eye*, 50.

61. Burton, *Anatomy of Melancholy*, i. 250–251.

62. Lavater, *Of Ghostes and Spirites*, 48–49.

63. Willis, *Two Discourses*, 94.

64. Levack, *The Devil Within*, 113–129; MacDonald (ed.), *Witchcraft and Hysteria*.

65. Jorden, *A Briefe Discourse*, 12–13. On the relationship between dreaming and insanity see Pigman III, *Conceptions of Dreaming*, 190–192. On the various states of sleep in early modern theory see the recently edited volume Simpson-Younger and Simon, *Forming Sleep*, esp. introduction.

66. Cotta, *A Short Discoverie*, 61–64.

67. Bernard, *Guide to Grand-jury Men*, 16–18; and Norton, *Devil’s Snare*, 30.

68. On the role of physicians in distinguishing natural from preternatural phenomena, see Gevitz, “The Devil Hath Laughed”; and Bonzol, “The Medical Diagnosis of Demonic Possession”.

69. *The Athenian Oracle*, i. 280–281.

70. Bureau d’Adresse, *A General Collection of Discourses*, 200. For background to Théophraste Renaudot’s conferences held at the Bureau d’Adresse see Wellman, *Making Science Social*.

71. Casaubon, *A Treatise Concerning Enthusiasme*, 34.

72. Casaubon, *A Treatise Concerning Enthusiasme*, 37–47.

73. Agrippa, *Three Books of Occult Philosophy*, 492–493.

74. On the astral body and its influence on the natural magic tradition see Walker, *Spiritual and Demonic Magic*, 38–40.

75. Peter Marshall has found fleeting references to ghosts as astral projections in English sources: Marshall, *Beliefs and the Dead*, 249.

76. Ross, *Arcana Microcosmi*, 133–134.

77. Croll, *Philosophy Reformed*, 67–68.

78. Hartlib, “Letter 21 June 1657”, fol. 21A; Hartlib, “Letter 28 May 1657”, fol. 7A-7B. The belief that alchemical knowledge could be acquired through dreams was common amongst prominent exponents of natural magic, such as John Dee and Simon Forman: see Levin, *Dreaming the English Renaissance*, 39, 57.

79. Hartlib, “Letter 8 June 1657”, fol. 19B. On the distinction between soul and spirits, see Schaffer, “Godly Men and Mechanical Philosophers”.

80. Kassell, “Magic, Alchemy and the Medical Economy”; Dobbs, “Natural Philosophy of Sir Kenelm Digby”; and Debus, “Robert Fludd”. On Van Helmont’s view of nature and his clash with scholasticism see Waddell, “The Perversion of Nature”.

81. On Van Helmont’s anthropology see Hedesan, *An Alchemical Quest*, 137–166.

82. Van Helmont, *Works*, 337.

83. Van Helmont, *A Ternary of Paradoxes*, 59–60, on sleepwalking see also 54.

84. Hartlib, “Letter, 3 November 1657”, fol. 14B.

85. Hartlib, “the art of interpreting dreams”, fol. 10A-13A. Beale gives a number of examples of premonitory dreams experienced by himself and others in Hartlib, “Letter, 28 May 1657”.

86. Handley, “Sleepwalking, Subjectivity”, 317.

87. Pigman III, *Conceptions of Dreaming*, 195–198.

88. Schott, “Van Helmont on Imagination”.

89. Koslofsky, *Evening’s Empire*, ch. 3.

90. Hunter, *The Decline of Magic*, ch. 6.

## Websites

American Academy of Sleep Medicine. http://sleepeducation.org/
